# Factors determining the smooth flow and the non-operative time in a one-induction room to one-operating room setting

**DOI:** 10.1111/jep.12288

**Published:** 2014-12-11

**Authors:** Jan P Mulier, Liesje De Boeck, Michel Meulders, Jeroen Beliën, Jan Colpaert, Annabel Sels

**Affiliations:** 1 Department of Anesthesiology, Intensive Care and Reanimation, Department of Anesthesiology, AZ Sint-Jan Brugge-OostendeBruges, Belgium; 2 Department of Cardiovascular Sciences, Anesthesiology and Algology, KU LeuvenLeuven, Belgium; 5 Faculty of Economics and Business, Research Centre for Quantitative Business Processes, KU LeuvenCampus Brussels, Brussels, Belgium; 6 Faculty of Economics and Business, Centre for Globalization, Innovation and Competition, KU LeuvenCampus Brussels, Brussels, Belgium

**Keywords:** anaesthesia induction room, non-operative time, operating room management, operating room scheduling, parallel flow in a surgery line

## Abstract

**Rationale, aims and objectives:**

What factors determine the use of an anaesthesia preparation room and shorten non-operative time?

**Methods:**

A logistic regression is applied to 18 751 surgery records from AZ Sint-Jan Brugge AV, Belgium, where each operating room has its own anaesthesia preparation room. Surgeries, in which the patient's induction has already started when the preceding patient's surgery has ended, belong to a first group where the preparation room is used as an induction room. Surgeries not fulfilling this property belong to a second group. A logistic regression model tries to predict the probability that a surgery will be classified into a specific group. Non-operative time is calculated as the time between end of the previous surgery and incision of the next surgery. A log-linear regression of this non-operative time is performed.

**Results:**

It was found that switches in surgeons, being a non-elective surgery as well as the previous surgery being non-elective, increase the probability of being classified into the second group. Only a few surgery types, anaesthesiologists and operating rooms can be found exclusively in one of the two groups. Analysis of variance demonstrates that the first group has significantly lower non-operative times. Switches in surgeons, anaesthesiologists and longer scheduled durations of the previous surgery increases the non-operative time. A switch in both surgeon and anaesthesiologist strengthens this negative effect. Only a few operating rooms and surgery types influence the non-operative time.

**Conclusion:**

The use of the anaesthesia preparation room shortens the non-operative time and is determined by several human and structural factors.

## Introduction

Operating room (OR) management has been a major topic of research in the last decade [1]. This is not surprising as ORs generate considerable costs and revenues in a hospital [2],[3]. Many of the studies rely on advanced planning and scheduling techniques to optimize OR performance. Those techniques use deterministic or stochastic data as input. The impact of variability is determined *ex ante* (i.e. beforehand) in the stochastic case and might be introduced *ex post* (i.e. afterwards) in the deterministic case to verify the robustness of the results. In either case, the variability is assumed to be known (i.e. it is set at a certain level). In general, it is very well known that variability degrades performance in some way [4],[5]. As such, intervention is required, and can take the form of decreasing and/or coping with variability. Coping boils down to protecting the system against variability and vulnerability. This can take place in three ways: by an inventory buffer (safety stock), by a capacity buffer (excess capacity) and/or by a time buffer (safety time) [4]. Decreasing the variability is a second intervention measure. It is frequently encountered in practice. An appointment system, for instance, aims at reducing the random variability that is a natural part of customer arrivals. Appointment systems are very common in health care.

In studies related to OR management, only limited attention is paid to the factors causing this variability. However, identifying the causes of variability might help to decrease variability, and thus to improve OR performance, even before any optimization takes place. OR performance is often measured in terms of non-operative time (NOT), that is the time between the start of a surgery and the end of the surgery of the previous patient, within regular working hours. The smaller this NOT, the higher the OR efficiency, and the higher the probability to allow the addition of new operative cases on the same day. In order to decrease the NOT, some hospitals started to remove some activity not essential for the OR to another room. This led to an increase in the number of stages in a surgery line and a reduction of the time spent in a stage. The anaesthetic preparation and induction is an example of an activity that can be done in an anaesthesia induction room (IR) outside, but close to the OR. The anaesthesia induction phase is decoupled from the surgery phase and becomes a new stage 6–11. Friedman [12] calls this parallel processing although no new line is opened. In such a process, the next patient is induced while the previous patient is being operated. Marjamaa [11] clearly demonstrates that a process redesign in the OR by performing certain activities in different stages can significantly reduce NOT. But, even in such a setting, a smooth succession of patients is not always guaranteed, especially if no more manpower is available and the same quality and safety goals are kept. This leads to the goal of the paper, searching for factors hampering this smooth flow in order to identify the variability factors that can be decreased or should be coped with.

The literature presents different studies related to OR improvements, but they all differ from the study we will present here. Besides the fact that most studies rely specifically on the sequential setting of anaesthesiology and surgery in the same stage, all studies aim at streamlining the steps within and/or between preoperative, intraoperative or post-operative patient care [12] in some way. They focus on, for example:

- the anaesthesiology part 13–15;
- the inefficiency (also denoted as ‘muda’ or wasteful activities) within the perioperative workflow 16–18;
- OR teamwork, OR management tools, communication and ‘learning effects’ of different OR actors 19–25; and
- parallelization within the sequential structure of patients moving through the operating theatre (OT) [12],[26].

This separate induction-preparation room for anaesthesia is not always used. In case of manpower shortage, late arrival of the patient or medical reason, the separate use of an anaesthesia preparation room might be abolished. In this paper, we will focus on the main variability factors constraining the smooth flow in a separate anaesthesiology and surgery stage. As far as we know, this has not yet been investigated.

## Methods

### Data

This paper uses real-life data from the AZ Sint-Jan Bruges (Belgium) hospital in which induction and surgery occur at different stages. Permission was given by the Institutional Review Board to analyse the records anonymously. Each OR has its own adjacent IR. The data contain all surgeries performed in 2010, which amounts to 18 751 records in total. Information for each record (surgery) is available and is listed in Table 1.

**Table 1:** The data for each surgery record

Data	Specification
Date	The date on which the surgery took place
Operating room	The operating rooms where the surgery took place
Surgery type	A detailed description of the surgery type
Scheduled duration surgery	The scheduled duration of the surgery
Elective or not	Whether the surgery was an elective or a non-elective one
Surgeon	The surgeon who operated the patient
Anaesthesiologist 1	The (supervising) anaesthesiologist who induced the patient
Anaesthesiologist 2	The resident anaesthesiologist (if applicable)
Arrival operating theatre	The arrival time at the operating theatre
Arrival induction room	The arrival time at the induction room
Start induction	The starting time of the induction
Arrival operating room	The arrival time at the operating room
Start surgery	The starting time of the incision
End surgery	The ending time of the incision

The OT is defined as the area where preoperative, intraoperative and post-operative patient care takes place. The records are then sorted according to the following priority: date, OR and the arrival time at the OR. This enables the computation of the NOT for each record (if applicable). The NOT is defined as the time between wound closure of the previous surgery and the incision of the next surgery (that takes place in the same OR on the same day). This time is used for wound dressing, waking up of the patient, transferring to the post-anaesthestic care unit (PACU), cleaning of the room, installation of the next patient and anaesthesia induction if not done yet, clipping, surgical positioning, prepping and draping, surgery table preparation, and surgeon preparation.

In a teaching hospital such as AZ Sint-Jan Bruges, a limited number of anaesthesias are performed by a resident in training under supervision of an anaesthesiologist. All cases performed by residents under supervision are grouped together under the name residents while all other cases are classified under a specific anaesthesiologist.

The smoothness of the flow is mainly determined by the succession of the patient flow undergoing surgery and their successors being induced. In order to ‘quantify’ this flow, we start our analysis by dividing the patients in different groups. These groups are defined by means of two criteria:

- where the patient is situated in its surgery process at the moment the preceding patient's surgery has ended; and
- where induction and/or surgery take place (either in the IR or in the OR).

**Figure 1:**
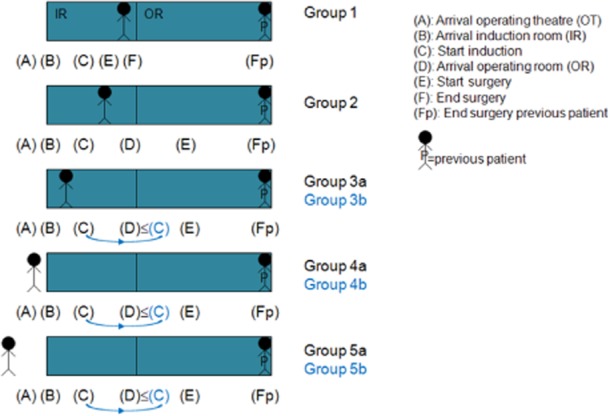
The different patient groups.

The groups are represented in Fig. 1.

Besides the first surgeries in row on a given day and in a given OR (group 0), a patient can be situated as follows in its surgery process when the previous patient's surgery is finished (see Fig. 1):

- the patient's surgery has already started (group 1);
- the patient's induction has already started (group 2);
- the patient has already arrived at the IR, but induction has not yet started (group 3);
- the patient has already arrived at the OT, but not at the IR (group 4); and
- the patient has not yet arrived at the OT (group 5).

Patients belonging to group 1 are induced and operated in the IR, and these are mainly very short procedures for which the operating equipment is not needed; the patients of group 2 are induced in the IR and operated in the OR; and the patients of groups 3, 4 and 5 can be induced in the IR (the ‘a’ group) or the OR (the ‘b’ group), and are operated in the OR. Groups 3, 4 and 5 represent 58% of the records as compared with the total number of records for groups 1–5. This result is in line with Saha [27] who proved that considerable operating time is wasted while patients are being transferred to the OT.

It is clear that for the analysis of the value of an extra stage, only groups 2, 3a, 4a and 5a are of interest. That is because those records represent the normal setting of induction in the IR and surgery in the OR. Further on, we only included records belonging to normal working hours within weekdays, and records with ORs, surgeons, anaesthesiologists and surgery types having at least 20 records. The remaining records make up a set of 5048 records.

In what follows, we refer to a group or bundle of groups as ‘G’ followed by the number of the group(s). For example, G2 represents the records belonging to group 2; G3a4a5a represents the records belonging to groups 3a, 4a and 5a. We notice that G2 has significantly lower NOTs as compared with groups 3a, 4a and 5a. The average NOTs and the corresponding 95% confidence intervals for G2, G3a, G4a and G5a are represented in Table 2.

**Table 2:** The average non-operative times and the corresponding 95% CI for G2, G3a, G4a, and G5a

Group	Lower bound 95% CI NOT (in min)	Mean NOT (in min)	Upper bound 95% CI NOT (in min)
G2	16.09	16.45	16.81
G3a	26.12	26.77	27.42
G4a	40.73	43.17	45.61
G5a	56.77	65.64	74.52

CI, confidence interval; NOT, non-operative time.

**Figure 2:**
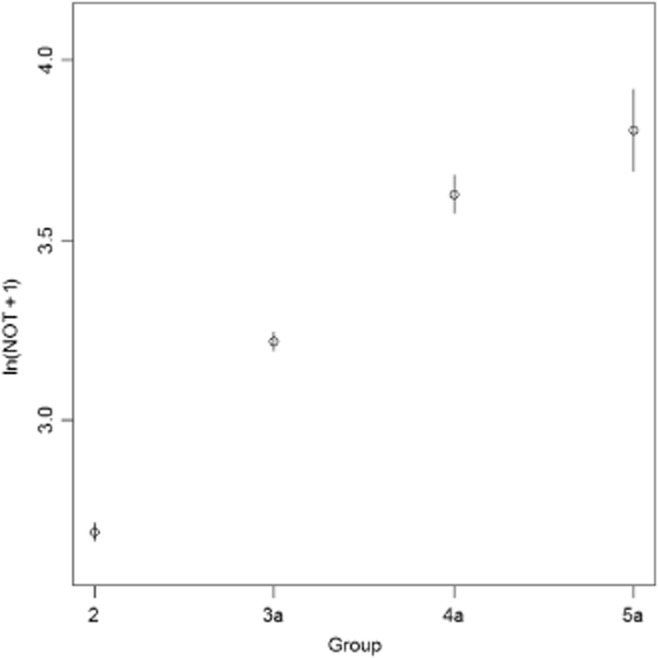
The average and 95% confidence intervals of ‘ln(NOT + 1)’ of groups 2, 3a, 4a, and 5a. NOT, non-operative time.

However, as the distribution of NOT tends to be very skewed, and contains many zero values, the normality assumption of analysis of variance is not fulfilled. That is why we will use ‘ln(NOT + 1)’ and analyse if this variable differs significantly among the different groups. This transformation to ‘ln(NOT + 1)’ reduces the influence of outliers, and yields an approximately normal distribution. Figure 2 clearly demonstrates that the average of ln(NOT + 1) is significantly increasing for groups 2, 3a, 4a and 5a. We also note that 44% of the 5048 records belong to G3a4a5a.

In what follows, we will present all models.

### Logistic regression model

In order to find out the variability factors constraining the smooth flow within the OT, a dummy dependent variable will be defined by dividing the records into two groups: we bundle the records of groups 3a, 4a and 5a versus the records of group 2. More specifically, the variable *Y*_i_ equals 1 if surgery i belongs to G3a4a5a and 0 if it belongs to G2. Furthermore, *x*_i_ represents a vector of *P* covariate values that describes surgery i (e.g. surgery type, OR, etc.). When using logistic regression, the binary outcome variable *Y*_i_ given *x*_i_ is modelled as the sum of the conditional mean E(*Y*_i_|*x*_i_) and an error term ε_i_ as represented in Eq. (1).


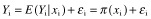
(1)

The logit of the conditional mean π(*x*_i_) is assumed to be a linear function of *P* covariates *x*_ip_ (*P =* 1,..,*P*):


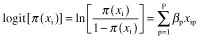
(2)

Furthermore, if *y*_i_ = 1, it is assumed that ε_i_ equals 1 – π(*x*_i_) with probability π(*x*_i_), and if *y*_i_ = 0, it is assumed that ε_i_ equals −π(*x*_i_) with probability 1 − π(*x*_i_). The latter assumptions about ε_i_ imply that, conditional on *x*_i_, the outcome values *y*_i_ are independently and identically Bernoulli distributed with probability 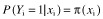:


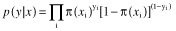
(3)

In sum, the logistic regression model in Eq. (2) uses a linear regression model to predict the logit of the probability that a surgery i with covariate values *x*_i_ will be classified into one as opposed to the other of the two categories of the dependent variable (i.e. G3a4a5a versus G2).

For the right-hand side of Eq. (2), we will use the following predictors:

- the OR in which surgery took place (categorical variable with 13 categories);
- the actual surgeon (categorical variable with 38 categories);
- the actual anaesthesiologist (categorical variable with 22 categories);
- the surgery type (categorical variable with 76 categories) (see also [28] for inclusion of this predictor);
- whether the current surgery is elective or not [binary variable: non-elective (1) versus elective (0)];
- whether the previous surgery was elective or not [binary variable: non-elective (1) versus elective (0)];
- a surgeon switch [binary variable: yes (1) versus no (0)];
- an anaesthesiologist switch [binary variable: yes (1) versus no (0)];
- the scheduled duration of the previous surgery (continuous variable);
- whether both the current surgery and the previous surgery where elective [binary variable: both elective (1) versus otherwise (0)]; and
- the interaction effect of a surgeon and anaesthesiologist switch [binary variable: both a switch of surgeon and anaesthesiologist (1) versus otherwise (0)].

As the actual surgeon and anaesthesiologist equal the scheduled ones for all surgeries scheduled at latest the day before the actual surgery, we will use the actual surgeon and actual anaesthesiologist. Only for urgencies (i.e. surgeries scheduled on the day of surgery, which are part of the group of non-elective surgeries), there is a switch in surgeon which amounts to 5% of all surgeries. A surgery is defined as elective (non-elective) if it is scheduled the latest at (after) 1200 h the day before surgery. We note that the scheduled duration of the previous surgery is the time between the scheduled start and the scheduled end of the incision of the previous surgery (i.e. the non-surgical time is not included). As all nurses and medical personnel are dedicated to a specific OR, we may assume statistical independence among ORs (as opposed to e.g. [29],[30] ). This personnel performs almost all tasks (cleaning, moving patients, bring along all material, etc.). Only floor cleaners are shared among roomers, but if those are not available when needed, the dedicated nurses perform this task. As a result, we may assume statistical independence of NOT among ORs on the same day at approximately the same time of the day. Moreover, a surgeon is never scheduled in more than one OR simultaneously.

Furthermore, we use effect coding for each of the categorical variables. This means that the regression coefficients associated to the categories of a categorical variable sum to zero, and that the regression coefficient of a category *c* indicates how much higher logit *P*(*Y*_i_ = 1|*x*_i_) is for category *c* as compared with the average effect across all categories of the categorical variable.

To build the models we apply a forward variable selection procedure on all the available predictors. The effect coding variables used to model a particular categorical variable are considered as a block in this variable selection procedure, that is, they are all either included or excluded. Finally, we note that the surgery type and the surgeon could not be included in the same model, as this yields unstable parameter estimates for part of the regression coefficients associated to these categorical variables. To investigate the effect of each of these variables on the results of the analysis, we will therefore build two models: one that includes the surgeon and one that includes the surgery type.

Finally, we note that by taking the exponent in Eq. (2), the odds for being in G3a4a5a rather than G2 is modelled as a multiplicative function of the predictor variables:


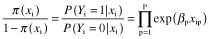
(4)

Eq. (4) implies that the exponentiated regression coefficients can be interpreted in terms of the odds. More specifically, for a binary (0/1) predictor *X* with regression coefficient β, if all other predictors in the model remain constant, the odds of being in G3a4a5a rather than in G2 are exp(β) larger if *X* equals 1 than if *X* equals 0. Furthermore, for category *c* of an effect-coded categorical variable *X*, a regression coefficient β means that, if all the other predictors remain constant, the odds are exp(β) times larger for category *c* than for the geometric mean of the odds of all the categories of *X*.

### Log-linear regression model

As OR performance is often measured in terms of NOT, we perform an additional analysis. In this analysis, ‘ln(NOT + 1)’ will be the dependent variable. Note from the beginning of this section that the traditional assumptions of linear regression are not fulfilled as the distribution of NOT tends to be very skewed, and contains many zero values. That is why ‘ln(NOT + 1)’ will be used. Similar to the logistic regression analysis, the log-linear model is built by applying a forward variable selection procedure to all available predictor variables. By doing so, we can compare the results of the logistic regression and log-linear regression model.

The log-linear model is then as follows:


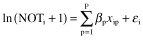
(5)

The error terms ε_i_ are assumed to be independently and identically normally distributed with mean 0 and variance σ^2^. To interpret the effects in this model on the scale of the original variable (NOT + 1) we will use the fact that a unit increase in variable *X*_p_ increases the Median (NOT + 1) with a factor exp(β_p_). For an effect-coded categorical variable *X* a regression coefficient of β for category *c* means that the median (NOT + 1) is exp(β) times larger than the geometric mean of median (NOT + 1) of all categories.

Note that, in the present study, we are mainly interested to investigate how explanatory variables affect the median NOT rather than the average NOT as the former is a more robust measure that is less influenced by outliers. Using the log-linear model, inferences about median NOT are naturally supported. However, if inferences about the average NOT were of primary interest, an adapted statistical analysis would be necessary [31],[32].

## Results

In what follows, we will present the main results for all four models (both the logistic regression models with surgery type and surgeon and both the log-linear regression models with surgery type and surgeon). The detailed results will only be presented for the models with surgery type. The reason for presenting the models with surgery type more in detail is because these models yield the best fit both for the log-linear analysis and the logistic regression analysis. More specifically, the log-linear model with the surgery type clearly has a better fit (higher *R*^2^_adj_ = 0.225; see also infra) than the model with surgeon (*R*^2^_adj_ = 0.188). Furthermore, although the logistic regression model with surgeon has a better balance between complexity and fit than the model with surgery type (i.e. Bayesian information criterion values of 6097 and 6517, respectively), we prefer to discuss the logistic model with surgery type in more detail, as this model yields more accurate predictions of the observed proportion of surgeries in G3a4a5a across the entire range of the probability scale (see Fig. 5). Finally, a Hosmer–Lemeshow goodness-of-fit test [33,34] using 20 groups shows that the model with surgery type fits the data rather well [χ^2^ = 15.5, degrees of freedom (df) = 18, *P =* 0.629] whereas the model with surgeon does not fit the model in an absolute sense (χ^2^ = 37.1, df = 18, *P* < 0.01).

We only present those exponentiated coefficients of all models that differ significantly from zero using significance levels of 5%. Note that the coefficients of all the categorical variables are evaluated on a ‘5% divided by the number of categories’ significance level. For example, as there are 13 ORs, coefficients will be tested on a significance level of 5%/13 (=0.003846). This division by the number of categories (which is equivalent to using a Bonferroni correction) ensures that the type I error for the whole of statistical tests on a categorical variable does not exceed the 5% level. In addition, in the logistic regression analysis the standard errors (and consequently the *P*-values) were adapted to account for overdispersion. Such overdispersion may for instance result from dependencies among observations that are not taken into account in the analysis. To correct for overdispersion, the standard errors of the regression coefficients were multiplied with the square root of the estimated dispersion parameter. For models with surgery type and surgeon, the deviance-based dispersion parameter (computed as the model deviance divided by the df of the model) equals 1.13 and 1.12, respectively. Furthermore, the Pearson chi-squared based dispersion parameter (computed as the Pearson chi-squared statistic divided by the df of the model) equals 1.03 and 1.02 for both models, respectively. We may conclude that there is little evidence for overdispersion. As the deviance-based overdispersion parameter was slightly higher than the dispersion parameter based on the Pearson chi-squared statistic, we used the former to correct the standard errors of logistic regression coefficients.

We first show the results of the logistic regression model. Thereafter, we present the results of the log-linear regression model. Finally, we discuss the general performance of both models.

### Results of the logistic regression model

**Figure 3:**
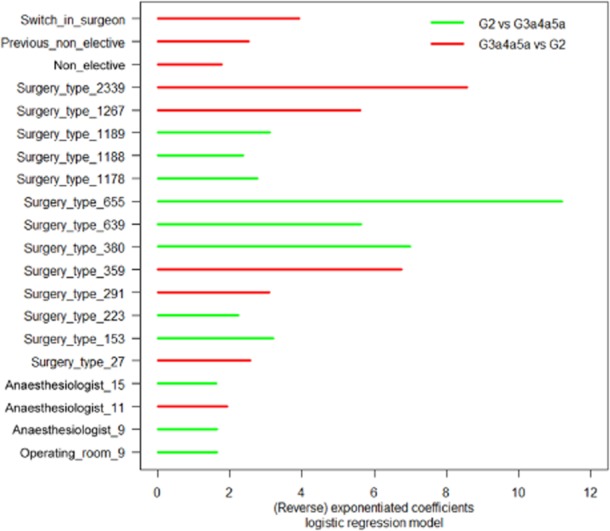
Significant (reverse) exponentiated coefficients of the logistic regression model.

We start with the results of the logistic regression model. Note that an exponentiated coefficient of one (or a regression coefficient equal to 0) leaves the odds (of an occurrence in G3a4a5a versus G2) unchanged, an exponentiated coefficient greater than one (or a positive regression coefficient) increases the odds, and an exponentiated coefficient smaller than one (or a negative regression coefficient) increases the reverse of the odds (i.e. 1/odds). The exponentiated coefficients greater than or equal to one and the reverse of the exponentiated coefficients (when the exponentiated coefficients are smaller than one) that differ significantly from zero are represented in Fig. 3.

In Fig. 3, the values of the exponentiated coefficients (red bars) show how many times more probable it is to be classified in G3a4a5a versus G2. The values of the reverse of the exponentiated coefficients (green bars) show how many times more probable it is to be classified in G2 versus G3a4a5a. This classification is:

- as compared with the geometric mean of the odds of all the categories for the categorical predictors (e.g. for surgery type_655 the odds of being classified in G2 rather than in G3a4a5a, are 11.2 times larger than the (geometric) average odds for all the categories of surgery type);
- for a one-unit increase in the continuous predictor (the continuous predictor is not significant in the model); and
- as compared with the ‘zero'-valued reference category for the binary predictors (e.g. for non-electives the odds of being classified in G3a4a5a versus G2, are 1.8 times larger than for electives).

We now give an overview of the main results of the logistic regression models (i.e. the model with surgery type and the model with surgeon). The results for the latter model are given between brackets.

The analysis shows that 13 (16) out of 76 (38) analysed surgery types (surgeons) have an independent influence on doing more or less cases with an anaesthesia induction before end of surgery, while only 3 (the same anaesthesiologists for both models) out of 22 analysed anaesthesiologists have an independent influence on doing more or less cases with an anaesthesia induction before end of surgery. For the surgery type (surgeon) with maximum positive influence, it is 11.2 (7.7) times more probable to use the IR as compared with the average surgery type (surgeon) while for the anaesthesiologist with maximum influence, it is only 1.7 (1.8) times more probable to use the IR as compared with the average anaesthesiologist. For the surgery type (surgeon) with maximum negative influence, it is 8.6 (6.4) times more probable not to use the IR as compared with the average surgery type (surgeon) while for the anaesthesiologist with maximum negative influence, it is only 1.9 (1.9) times more probable not to use the IR as compared with the average anaesthesiologist.

Although most surgery types take place in a limited number of rooms, the room number itself has almost no influence on using more the IR, except for room 9 with frequent bariatric surgery. In the model with surgeons, no OR is significant.

Further on, both models report on a negative influence of switches in surgeons, surgeries being non-elective and previous surgeries being non-elective on doing more or less cases with an anaesthesia induction before end of surgery.

We notice that the effects of the same predictors for both models are equivalent (negative or positive in both models), but in the model with surgeons, all significant effects are larger for those predictors.

### Results of the log-linear regression model

**Figure 4:**
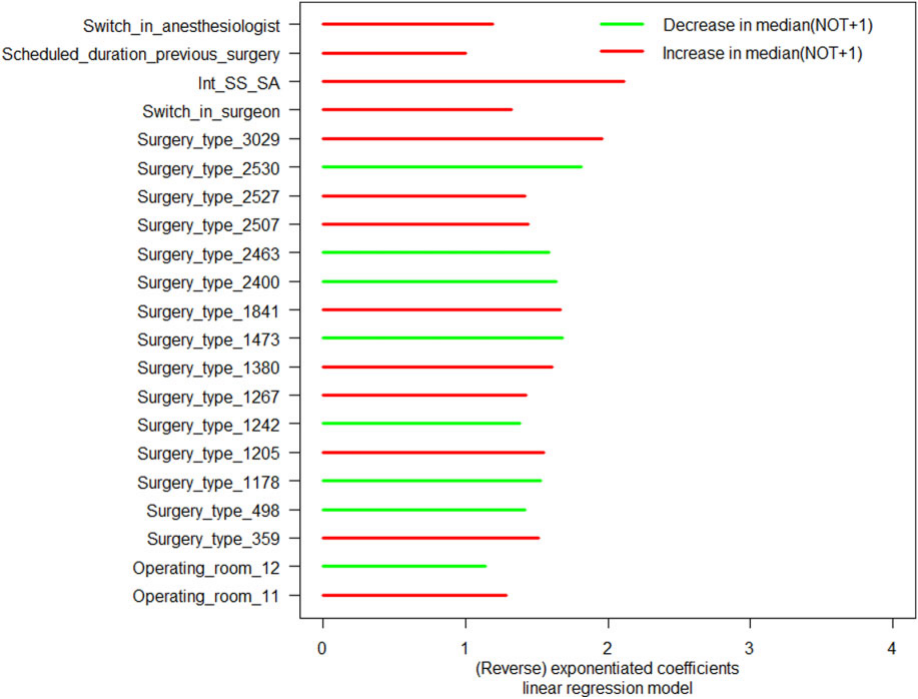
Significant (reverse) exponentiated coefficients of the linear regression model.

In presenting the results of the log-linear regression models, we also opt for the exponentiated coefficients. An exponentiated coefficient of one leaves ‘NOT + 1’ unchanged, an exponentiated coefficient greater than one increases ‘NOT + 1’ and an exponentiated coefficient smaller than one decreases ‘NOT + 1’. In the latter case, we will use the reverse of the exponentiated coefficient to indicate how many times ‘NOT + 1’ becomes smaller. The exponentiated coefficients greater than or equal to one, and the reverse of the exponentiated coefficient (when the exponentiated coefficients are smaller than one) that differ significantly from zero are represented in Fig. 4.

**Figure 5:**
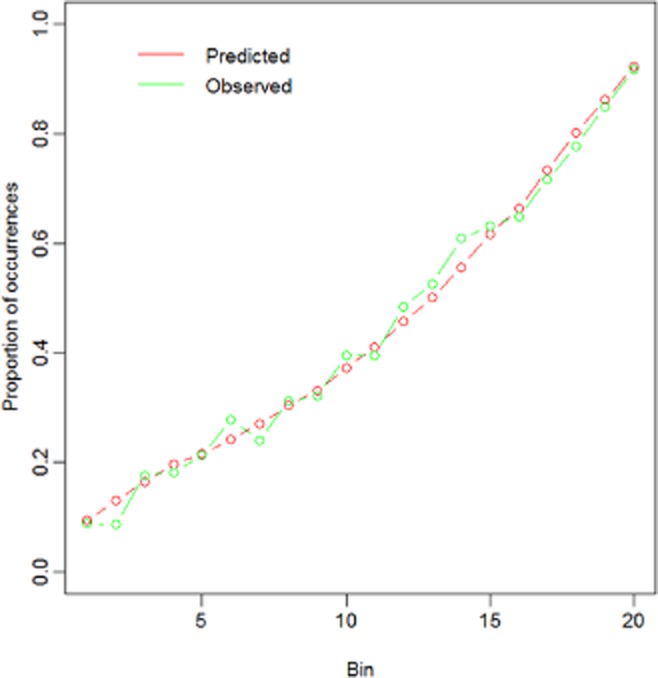
Predicted and observed proportions of occurrences in G3a4a5a of the logistic regression model with surgery type.

In Fig. 4, the values of the exponentiated coefficients (red bars) show how many times median (NOT + 1) increases. The values of the reverse of the exponentiated coefficients (green bars) show how many times median (NOT + 1) decreases. This increase/decrease is:

- as compared with the geometric mean of median (NOT + 1) of all categories for the categorical predictors [e.g. median (NOT + 1) for surgery type_2530 is 1.8 times smaller than the (geometric) average median (NOT + 1) for all surgery types];
- for a one-unit increase in the continuous predictor [e.g. a 1-min increase in the duration of the previous surgery makes median (NOT + 1) 1.0004 times larger]; and
- as compared with the ‘zero'-valued reference category for the binary predictors [e.g. surgeries with a switch in anaesthesiologist have a median (NOT + 1), which is 1.2 times higher than surgeries without switch in anaesthesiologist].

Figures 3 and 4 clearly indicate that analysing factors causing NOT are partly different from the factors hampering the smooth flow in a surgery line with an anaesthesia preparation room.

We now give an overview of the main results of the log-linear regression models (i.e. the model with surgery type and the model with surgeon). The results for the latter model are given between brackets.

Operating room_12 (_0) has shorter NOT times than the average OR; operating room_11 has longer NOT times as compared with the average OR. None of the anaesthesiologists have shorter or longer NOT times as compared with the average anaesthesiologist. Seven (seven) surgery types (surgeons) have shorter NOT times and one (five) of them use the IR more than the average surgery type (surgeon). Eight (six) surgery types (surgeons) have longer NOT times and two (four) of them use the IR less than the average surgery type (surgeon). All other factors like a switch in surgeon or anaesthesiologist, a switch in both surgeon and anaesthesiologist, a non-elective of the case before (only for the model with surgeon), and the duration of the previous surgery increase NOT.

### Evaluation of the models' performance

We evaluate the performance of the logistic regression model (with surgery type) by comparing the observed proportion of occurrences in G3a4a5a with the proportion predicted by the model. To this end, we sort all records in ascending order of predicted probability and divide them in 20 adjacent equally sized bins. The predicted proportion of occurrences in G3a4a5a for a bin is computed as the average predicted probability for the bin. Figure 5 displays the observed and predicted proportion of occurrences of interest per bin.

Figure 5 shows that the observed and predicted proportion of occurrences is very close to one another. Furthermore, a Hosmer–Lemeshow goodness-of-fit test [33,34], which evaluates whether expected proportions deviate significantly from observed proportions, indicates that the model fits the observed proportions rather well (*X*^2^ = 15.5, df = 18, *P =* .629).

Figure 5 also clearly demonstrates the predictive power of the logistic regression model as compared with random allocation. For instance, for the top 5% of records with the highest predicted probability (i.e. bin 20), the observed proportion of occurrences is about 2.1 times larger than the observed proportion of occurrences in the entire subset (i.e. 44% of occurrences in G3a4a5a). In other words, for the top 5% of records with the highest predicted probability, the observed proportion of occurrences goes up to 92%.

The log-linear regression model (with surgery type) is evaluated using *R*^2^_adj_. This performance measure equals 0.225.

## Discussion

Our results indicate that the occurrence of surgeries in groups 3a, 4a and 5a (G3a4a5a) versus group 2 (G2) is not a random process. Along the same lines, NOT is not exclusively attributable to natural variability. In other words, artificial variables cause this categorization/NOT. We relate this finding to the general concept of artificial variability in hospitals (as opposed to natural variability). Note that we were not able to analyse some factors like the missing manpower in surgeons, anaesthesiologists or nurses for some cases, or the missing equipment or logistics for other cases. Therefore, we consider the following artificial causes under the analysed factors.

- One important restriction is the fact that choosing for group 2 rather than for group 3 is also related to the amount of preparation work to induce anaesthesia and the work to wake up the patient. The surgery type determines this choice and therefore it influences more than the anaesthesiologist the chosen group. If group choice were entered at random, the difference in NOT within the different groups and the difference between the groups would become larger.
- The OR does not play a major role in the analysis. Only operating_room_9 has a positive effect with respect to surgeries being classified in G2 rather than G3a4a5a, operating_room_12 with respect to surgeries having less NOT, and operating room_11 with respect to surgeries having more NOT. Operating_room_9 has always a heavy program of general surgery. This stimulates everyone to work harder to be able to get all cases done in time. Therefore G2 is more frequent. The same is true for operating room_12, mainly used for short urology procedures. The urologic procedures of operating_room_12 have a limited surgical preparation time explaining the low NOT. Operating room_11 has very short procedures without preparation and long procedures with less pressure.
- The probability to be classified into G2 versus G3a4a5a is strongly determined by the surgery type. Some surgery types cause surgeries more to fall in the ‘good’ group (G2) while other surgery types tend to cause a delay in the streamlined process of operative care [i.e. there is a higher probability to belong to the ‘bad’ groups (G3a4a5a) ]. However, mostly other surgery types cause less/more NOT (except for surgery type_359, _1178 and _1167). Apart from these results being mainly attributable to the surgery type itself, other factors should be investigated as well. Does the surgeon request to transport the next patient in time and has the surgeon a strong influence on other persons to get ready in time? A possible explanation lies also in poor/good patient scheduling, as the surgeons are responsible for the daily patient schedules.
- A similar but less strong observation as described with the surgery type takes place with the anaesthesiologist. Only a few of them push surgeries more towards the ‘bad groups’ and vice versa. Here, experience or a more efficient way of working explains the results. Nevertheless only one anaesthesiologist performed less while all trainees supervised by anaesthesiologists in the same OR have been included. A second reason is that some experienced anaesthesiologists having their own room are also responsible for trainees in another room and this will lower their own room efficiency by instructing and helping the trainees with their cases. Reducing variability is therefore not easy if the latter factors are not taken into account. Furthermore, no single anaesthesiologist causes more or less NOT. The reason is that shortening the NOT requires not only the use of the IR, but also a rapid awakening, a short transfer to the PACU, an early entry in the OR, allowing the surgical preparation to start immediately and in the shortest time possible. NOT shortening is achieved therefore by a coordinated action of the anaesthesiologist and the anaesthesia nurse with the surgeon, the surgeon in training, the scrub nurse and the circulating nurse.
- Non-electives have a higher probability to be part of the bad groups. This is mainly the result of the unpredictable character of non-electives resulting in delays within the preoperative process. In general, non-electives are known to cause variability. While the schedule of electives aims at the most efficient utilization of the available resources (OR, staff, etc.), non-electives typically disturb this schedule. Thereby, the quality of care decreases (longer delays), while costs rise. It is therefore important to look for the correct allocation of the available resources between elective and non-elective surgeries. A separate OR for non-elective surgeries hence leads to a smoother patient sequence, but at the cost of an extra OR to be equipped (capacity buffer).
- Surgeries preceded by a non-elective surgery, also typically score ‘badly’ (i.e. a higher probability to be part of the bad groups). This effect clearly illustrates the impact of non-electives on succeeding surgeries. A lack of additional personnel for these non-electives as well as poor communication between the different OR actors of both surgeries is probably the main cause here.
- A switch in surgeon has a negative impact on the smooth operative flow, and a switch in surgeon or anaesthesiologist results in increased NOTs. A switch in both surgeon and anaesthesiologist for a specific surgery strengthens the latter negative effect. Although such changes frequently appear to be unplanned, switches should clearly be avoided. This result can be used to improve the master surgery schedule (which defines the OR block assignments to surgical groups). A schedule with fewer switches will be exposed to lower artificial variability.
- A longer scheduled surgery duration of a predecessor has a negative effect on NOT.

## Conclusions

To conclude, master surgery schedules should take care to combine surgery duration with anaesthesia preparation time of the next case, and reduce switches in surgeons and/or anaesthesiologists in order to be exposed to lower artificial variability. At the same time, one must investigate and eliminate the causes for surgery types performing worse than the average surgery type. Similarly, anaesthesiologists not being part of the good performing group should be encouraged to work more efficiently if not responsible for anaesthesiologists in training. By doing so, artificial variability will further decrease. Finally, non-electives should deserve special attention concerning efficient utilization of available resources within the operative flow because their negative influence in disturbing the elective schedule propagates through subsequent surgeries.
